# Stress, pain, and work affiliation are strongly associated with health-related quality of life in parents of 14–15-year-old adolescents

**DOI:** 10.1186/s12955-021-01913-7

**Published:** 2022-01-06

**Authors:** Gudrun Rohde, Sølvi Helseth, Hilde Timenes Mikkelsen, Siv Skarstein, Milada Cvancarova Småstuen, Kristin Haraldstad

**Affiliations:** 1 Department of Health and Nursing, Faculty of Health and Sport Sciences, University of Agder, Postbox 422, 4604 Kristiansand, Norway; 2 Department of Clinical Research, Sorlandet Hospital, Kristiansand, Norway; 3 Department of Nursing and Health Promotion, Faculty of Health Sciences, Oslo Metropolitan University, Oslo, Norway

**Keywords:** Stress, Pain, Work affiliation, Parents of adolescents, Health-related quality of life

## Abstract

**Background:**

For many adults, their role as a parent is a vital part of their lives. This role is likely to be associated with a parent’s health-related quality of life (HRQOL). The aim of this study was to explore the associations between gender, demographic and psychosocial variables, pain, and HRQOL in parents of 14–15-year-old adolescents.

**Methods:**

This was a cross-sectional study that included 561 parents. Data on demographic, psychosocial variables and pain were collected using validated instruments. HRQOL was assessed using the RAND-36. Data were analysed using univariate and hierarchical multiple linear regression analyses.

**Results:**

Four hundred and thirty-six (78%) mothers and 125 (22%) fathers with a mean age of 45 (SD = 5) years were included. Eighty-one per cent were married/cohabiting, 74% worked full time, and 50% had university education of more than 4 years. Almost one-third reported daily or weekly pain, and more than half (58%) reported using pain analgesics during the previous 4 weeks. Mothers reported significantly lower scores on self-efficacy, self-esteem and for all RAND-36 domains, including the physical component summary (PCS) and mental component summary (MCS) and experienced greater stress than fathers. Hierarchical regression analyses showed that working part-time (beta = 0.40) or full time (beta = 0.52) (reference: not working) had the strongest positive effect on PCS. Absence from work for > 10 days (beta = −0.24) (reference: no absence), short-term pain (beta = −0.14), chronic pain (beta = −0.37) (reference: no pain), and stress (beta = −0.10) had the strongest negative effects on PCS. High self-esteem (beta = 0.11) had the strongest positive effect, whereas stress (beta = −0.58) and absence from work for > 10 days (beta = −0.11) (reference: no absence) had the strongest negative effects on MCS.

**Conclusion:**

Mothers reported significantly lower scores on self-efficacy, self-esteem, and HRQOL, and experienced greater stress than the fathers. A high proportion of parents reported pain. Pain, stress, and low work affiliation were strongly associated with decreased HRQOL in parents. We recommend that parents of adolescents should be provided guidance about coping with pain and stress, and facilitation of a strong work affiliation because these seem to be important to parents’ HRQOL.

## Introduction

The United Nations Sustainable Development Goal number 3 is to ensure healthy lives and to promote quality of life (QOL) for all at all ages [1]. This goal has been incorporated into national guidelines and goals, health-care practice, and within the general population, and the goals are important outcomes in research studies. QOL is increasingly used as an outcome measure in different research settings, including clinical practice and population health surveys, in both adult and paediatric populations. In previous studies of the general adult population, demographic variables such as male gender, higher educational level [2, 3], belonging to a higher socio-economic group [4], high income, being married or cohabiting, and being employed [5] are associated with high health-related quality of life (HRQOL). By contrast, having pain [6], older age [7], long-term disease or health problems [8], and an unhealthy lifestyle [4] are associated with low HRQOL. In this context, HRQOL is defined as a multidimensional construct that includes the individual’s subjective perspectives on the physical, psychological, social, and functional aspects of health [8].

For many adults, their role as a parent is a vital part of their lives. This role may influence their HRQOL and may vary with the age of their child. Being a parent of an adolescent can be challenging because adolescence is a unique and complex developmental phase. Adolescence is characterized by significant physical, cognitive, and psychosocial changes that are related to self-identity, peer relationships, development of autonomy, and sexuality [9], all of which can influence parents’ HRQOL.

Parents can experience pain and high levels of stress, which may affect their HRQOL and ability as caregivers [10, 11]. Previous studies have shown that pain influences HRQOL negatively, especially in women [4, 12], and that parents’ pain and pain coping patterns may be adopted by their children and thereby influence HRQOL of both parents and their children [10, 13]. In parents of preschool children in the general population, low parental HRQOL is predicted by mental health problems, and families with at least one individual experiencing problems and in need of assistance, such as psychotherapy and parenting programmes, rated their HRQOL lower than families without these problems [14]. However, knowledge about HRQOL in parents of adolescents in the general population is scarce. Studying parents’ psychosocial and physical well-being is important since taking care of an adolescent requires support from parents [10, 15, 16]. In addition, parental pain, physical and mental health problems, and HRQOL seem to have an effect for generations [14].

To enhance and understand HRQOL in parents of adolescents better, and to initiate preventive actions, it is important to identify the factors associated with HRQOL in parents. Previous research has studied HRQOL in parents of adolescents in selected patient groups or a limited number of potential predictors of HRQOL in the general adult population [17, 18]. Our focus here was to examine HRQOL in parents of adolescents by analysing a wide range of potential predictive factors of HRQOL. Given the previous studies of HRQOL in the general adult population and lack of knowledge about which demographic and psychosocial factors are associated with HRQOL in parents of adolescents in the general population, our aim was to identify possible associations between gender, demographic and psychosocial variables, pain, and HRQOL in parents of 14–15-year-old adolescents.

## Methods

### Study sample and data collection

This cross-sectional study was a part of the “Start Young—quality of life and pain in generations” study conducted in two regions in Southern- and Eastern Norway, which has about 1.6 million inhabitants (30% of the total Norwegian population) and an adolescent population (aged 14–15 years) of about 37,000 [19]. Data presented in this paper are baseline measurements from a larger longitudinal study, aiming to follow adolescents and their parents for 2 and 4 years from the adolescents’ age of 14–15 years, an important transitional phase in their life. In Norway, transition from lower secondary- to upper secondary school normally involves a shift of school institutions for 16-year-old adolescents which may affect their HRQOL.

Schools that include grade 9 (aged 14–15 years) in elementary school were stratified according to region, rural or urban district, and school size. Details have been reported previously [19]. The schools varied in size and location from city to suburb and admitted adolescents from different sociocultural and economic backgrounds. The potential participants in this part of the study were one parent of 1663 adolescents in grade 9 from the participating schools, 561 of whom participated, giving a response rate of 34%. The parents responded to the web-based questionnaire at home.

The “Start Young—quality of life and pain in generations” study was reviewed by the Norwegian Centre for Research Data (Ref: 60,981), and the necessary approvals were obtained.

### Instruments

#### Demographic variables

The first part of the questionnaire included self-reported data on demographic variables such as gender, date of birth, marital status, education, household income, absence from work, and region. Differences between the regions were adjusted for in multiple analyses because a previous study showed a negative pattern within some psychosocial parameters in one of the regions [20].

#### Pain, HRQOL, self-efficacy, self-esteem, loneliness, and stress

*Pain* was measured using the Brief Pain Inventory (BPI) [21] and some questions from the Lübeck Pain-Screening Questionnaire (LPQ) [22]. The BPI measure pain occurrence, worst pain severity, pain inference, and number of pain locations. The BPI has well-established validity and reliability internationally and in Norway [21, 23]. Pain interference questions were completed by those who scored ≥ 1 on the “pain on average” question (indicating that they had pain) [23]. Respondents who rated ≥ 1 on the “pain on average” question of the BPI were given two follow-up questions from the LPQ about pain duration and pain frequency. The LPQ is a structured self-report questionnaire that evaluates the prevalence and consequences of pain [22]. The Norwegian LPQ has satisfactory feasibility, content, and face validity [24]. Two questions derived from the Norwegian “Pain, youth and self-medication study” (SUS) [25] were used to measure the intake of over the counter (OTC) analgesics. The respondents were first asked about OTC analgesic intake during the past 4 weeks; those who answered “yes” were asked about the frequency of intake.

To assess *HRQOL* we used the RAND-36 generic questionnaire that includes eight domains: general health, bodily pain, physical function, role limitations (physical), mental health, vitality, social function, and role limitations (emotional). The eight domains can be combined into physical and mental sum scales that reflect physical (physical component summary [PCS]) and mental (mental component summary [MCS]) health. The RAND-36 scales were scored according to published scoring procedures, and each scale was expressed using values from 0 to 100, with 100 representing excellent health [26–29]. The Cronbach’s α values in this study were 0.89 for mental health, 0.89 for bodily pain, 0.83 for general health, 0.87 for social function, 0.89 for physical function, 0.93 for role limitation physical, and 0.87 for role limitation emotional.

*Self-efficacy* was measured using a general self-efficacy (GSE) scale, consisting of 10 items [30, 31]. The scale was designed to measure a general sense of perceived self-efficacy and aimed to predict an ability to cope with daily demands and adaptation after a stressful experience. The instrument has a four-point scale from 1 (completely wrong) to 4 (completely right) and scores on each item are summed and divided by 10 into a GSE score ranging from 1 to 4. Higher scores indicate higher GSE levels. The questionnaire has been shown to be reliable and valid [31]. Cronbach’s α in this study was 0.89.

*Self-esteem* was measured using a short version of the Rosenberg Self-Esteem Scale (RSES) [32], in which respondents rate four statements on self-perceptions on a 4-point Likert scale ranging from 1 (strongly disagree) to 4 (strongly agree). Higher values indicate higher levels of self-esteem. The respondents’ scores on each item were summed up and divided by 4 to create an RSES score of 1–4. The questionnaire has been shown to be reliable and valid [33]. Cronbach’s α in this study was 0.73.

*Loneliness* was measured using the eight-item version of the revised UCLA Loneliness Scale (ULS-8) [34]. This instrument is a short version of the widely used 20-item revised ULS-20 [35]. ULS-8 uses a 4-point Likert scale with values ranging from “never” to “always”. The total score ranges from 8 to 32 points, and higher scores suggest a higher degree of loneliness [35]. The ULS-8 questionnaire was translated into Norwegian using standardized translation procedures and validated as part of this study [36]. Cronbach’s α in this study was 0.87.

*Stress* was measured using the Perceived Stress Questionnaire (PSQ) [37–39], which is a 30-item questionnaire referring to the past 4 weeks answered using a 4-point rating scale ranging from 1 (almost never) to 4 (almost always). The answers were recoded so that higher values indicated higher levels of perceived stress. The resulting PSQ total score was linearly transformed to a number between 0 and 1 using the equation PSQ = (raw value − 30)/90 [37]. The Norwegian version of the instrument has been shown to have good reliability and validity [39]. Cronbach’s α in this study was 0.87.

### Statistical analyses

Descriptive statistics are presented for all participants. Continuous variables are described as mean (SD), and categorical variables as counts and percentage. Crude associations between pairs of variables were assessed using the *t* test for continuous data or chi-square test for categorical data.

Associations between the two RAND-36 sum PCS and MCS scores as the dependent variables and selected possible predictive factors were analysed using multiple linear regression models. The selected independent variables were grouped into the following six blocks (B1–B6): B1, demographic variables; B2, self-efficacy; B3, self-esteem; B4, pain; B5, loneliness; and B6, stress. These variables are known theoretically as clinically relevant variables reported in previous HRQOL research [40]. To assess possible associations between HRQOL and the variables in each block, linear regression models were fitted separately for PCS and MCS. The strength of the associations between the variables in each block (B1–B6) and the dependent variables (RAND-36 PCS and MCS) was quantified in terms of the effect sizes (standardized beta), with an effect size of 0.1–0.3 considered small, 0.3–0.5 medium, and > 0.5 large [41].

To assess possible confounding and *adjusted* associations with HRQOL, hierarchical regression analyses were conducted (enter method) for PCS and MCS. The variables were entered into the regression analyses based on the six B1–B6 blocks described above. Six linear regression models (M1–M6) were fitted for the PCS and MCS sum scores by adding variables from a previous model (block) consecutively; later models always included all the variables from previous steps. This approach was chosen as our aim was to investigate how the effect of selected possible predictive factors might change when we add new variables/blocks. Further, we wanted to uncover possible interactions, e.g. some variables which are not statistically significant might reach the level of statistical significance when other variables are present in a multiple model. The strength of the associations between the variables in each model (M1–M6) and the dependent variables (PCS and MCS) was quantified in terms of the effect size and proportion of explained variance [42]. All tests were two-sided, and *P* values ≤ 0.05 were considered statistically significant. All analyses were considered exploratory, so no correction for multiple testing was performed. All analyses were performed using IBM SPSS Statistics (version 27).

## Results

### Characteristics of the sample

The socio-demographic characteristics of the sample are presented in Table 1. Among the 561 parents, 436 (78%) were mothers and 125 (22%) were fathers, and their mean age was 45 (SD = 5) years. Eighty-one per cent were married/cohabiting, 74% worked full time, and 50% of the parents had completed university education of ≥ 4 years. Almost one-third reported daily or weekly pain, and more than half (58%) reported using pain analgesics during the previous 4 weeks. Mothers reported significantly lower scores on self-efficacy and self-esteem, and experienced greater stress than fathers. However, loneliness and pain scores did not differ between mothers and fathers. Mothers reported significantly lower scores for all SF-36 domains, including the PCS and MCS scores (Tables 2, 3).

**Table 1:** Characteristics of the sample (N = 561), and comparison between the 426 women and 125 men

Demographic	All N = 561	Mothers N = 426	Fathers N = 125	*P* value
Age, years mean (SD)	45 (5)	45 (5)	47 (5)	< 0.001
*Living condition*				0.261
Married/cohabitating	457 (81%)	353 (81%)	104 (83%)	
Single	33 (6%)	30 (7%)	3 (2%)	
Divorced or separated	65 (12%)	49 (11%)	16 (13%)	
Widowed	6 (1%)	4 (1%)	2 (2%)	
*Education*				0.615
Compulsory education	4 (1%)	3 (1%)	1 (1%)	
Post-compulsory 1–3 years	22 (4%)	16 (4%)	6 (5%)	
Post-compulsory 3 years	50 (9%)	43 (10%)	7 (6%)	
Certificate of apprenticeship	61 (11%)	46 (11%)	15 (12%)	
University < 4 years	141 (25%)	105 (24%)	36 (29%)	
University ≥ 4 years	283 (50%)	223 (51%)	60 (48%)	
*Employment status*				< 0.001
Full time	414 (74%)	304 (70%)	110 (88%)	
Part-time	105 (19%)	95 (22%)	10 (8%)	
Not working	42 (7%)	37 (8%)	5 (4%)	
*Absence from work last 3 months*				0.261
None	372 (66%)	280 (64%)	92 (74%)	
1–4 days	124 (22%)	100 (23%)	24 (19%)	
5–7 days	17 (3%)	14 (3%)	3 (2%)	
8–10 days	5 (1%)	5 (1%)	0	
More than 10 days	43 (8%)	37 (9%)	6 (5%)	
*Household income (NOK)*				0.001
< 250,000	5 (1%)	4 (1%)	1 (1%)	
250,000–450,000	43 (8%)	39 (9%)	4 (3%)	
451,000–750,000	96 (17%)	78 (18%)	18 (14%)	
751,000–1,000,000	129 (23%)	112 (26%)	17 (14%)	
> 1,000,000	288 (51%)	213 (46%)	85 (68%)	

Categorical data are presented as number (%) and continuous variables as mean (SD). Chi-square tests were used to compare differences in categorical variables and independent *t* tests for continuous data

**Table 2:** Description of pain in the sample (N = 561) and differences between woman and men

	All N = 561	Mothers N = 426	Fathers N = 125	*P* value
*Having pain today*				0.297
Yes	56 (19%)	103 (24%)	24 (19%)	
No	240 (81%)	333 (76%)	101 (81%)	
Average pain score^a^	1.6 (1.8)	1.8 (1.9)	1.0 (0.5)	< 0.001
Pain interference, activity^b^	2.6 (2.2)	2.6 (2.2)	2.3 (2.0)	0.269
Pain interference, emotions^b^	2.7 (2.0)	2.8 (2.1)	2.4 (1.9)	0.147
*Pain duration*				0.010
No pain	223 (40%)	159 (37%)	64 (51%)	
≤ 3 months	110 (19%)	88 (20%)	22 (18%)	
> 3 months	228 (41%)	89 (42%)	39 (31%)	
*Pain analgesics in the past 4 weeks*				
Yes	326 (58%)	263 (61%)	62 (50%)	0.029
No	235 (42%)	172 (39%)	63 (50%)	
*Frequency of pain analgesics in the past 4 weeks*				0.635
Daily	26 (8%)	21 (8%)	5 (8%)	
Every week, but not daily	78 (24%)	78 (25%)	12 (19%)	
Less often than every week	219 (67%)	174 (66%)	45 (73%)	
No intake	3 (1%)	3 (1%)	0	
*Family pain*				0.032
Yes	230 (41%)	191 (44%)	39 (31%)	
No	269 (48%)	197 (45%)	72 (58%)	
Don’t know	62 (11%)	48 (11%)	14 (11%)	
*Chronic illness*				0.383
Yes	128 (25%)	101 (23%)	27 (22%)	
No	423 (75%)	329 (76%)	94 (75%)	
Don’t know	10 (2%)	6 (1%)	4 (3%)	

Categorical data are presented as number (%) and continuous variables as mean (SD). Chi-square tests were used to compare differences in categorical variables and independent *t* tests for continuous data)

^a^Range: 0–10, where 10 indicates pain as bad as can be imagined

^b^Range 0–10, where 10 indicates complete interference of pain

**Table 3:** Descriptive characteristics of HRQOL, self-efficacy, self-esteem, loneliness and stress (N = 561), and differences between women and men

	All N = 561	Mothers N = 426	Fathers N = 125	*P* value
*HRQOL*				
RAND-36 PCS^a^	52 (10)	51 (9)	53 (7)	0.002
RAND-36 MCS^a^	52 (8)	51 (8)	54 (7)	< 0.001
*RAND-36 eight domains*				
Bodily pain	78 (23)	77 (24)	85 (20)	< 0.001
General health	77 (19)	76 (20)	80 (15)	0.012
Physical function	93 (13)	93 (14)	96 (10)	0.016
Physical role function	84 (33)	82 (35)	90 (26)	0.003
Mental health	81 (13)	80 (13)	84 (11)	< 0.001
Vitality	64 (20)	62 (21)	70 (18)	< 0.001
Social function	87 (20)	85 (21)	93 (15)	< 0.001
Emotional role function	89 (28)	88 (29)	93 (23)	0.035
*Psychological factors*				
General self-efficacy^b^	3.3 (0.4)	3.3 (0.4)	3.4 (0.4)	0.007
Loneliness^c^	12.7 (4.2)	12.8 (4.4)	12.5 (4.1)	0.573
Stress^d^	0.28 (0.14)	0.29 (0.16)	0.24 (0.14)	0.006
Self-esteem^e^	33.4 (0.55)	3.44 (0.55)	3.31(0.55)	0.026

Independent *t* tests were used to compare mothers and fathers

^a^The score for the SF-36 ranges from 0 to 100, where 100 indicates a high HRQOL. PCS, physical component summary; MCS, mental component summary

^b^General self-efficacy: range 1–4, where higher values indicate higher levels of self-efficacy

^c^Loneliness: range 8–32, where higher values indicate higher levels of loneliness

^d^Stress: range 0–1, where higher values indicate higher levels of stress

^e^Self-esteem: range 1–4, where higher values indicate higher levels of self-esteem

### Crude associations between demographic variables, psychosocial variables, pain and HRQOL

Unadjusted linear regression analyses were used to identify possible associations between the selected variables (blocks) and HRQOL. The strength of the associations between the variables in each block (B1–B6) and the dependent variables (RAND-36 PCS and MCS) was described in terms of the effect sizes and proportion of explained variance (Table 4). Working both part-time and full time (reference: not working) and higher scores for self-esteem had the strongest positive effects on PCS, whereas pain (reference: no pain) and high stress level had the strongest negative effects. High self-esteem, high self-efficacy, and working full time (reference: not working) had the strongest positive effects on MCS, whereas high stress level, loneliness, and pain lasting more than 3 months (reference: no pain) had the strongest negative effects. The highest explained variance was for the block including demographic characteristics (23.9%) for PCS and the block including stress alone (53.8%) for MCS.

**Table 4:** Unadjusted associations between gender, demographic variables, psychosocial variables, pain and HRQOL examined by linear regression analyses^abc^, N = 561

	SF36-PCS						SF-36-MCS					
	B1	B2	B3	B4	B5	B6	B1	B2	B3	B4	B5	B6
Gender (Ref = father)	− 0.05						− 0.08					
Age	− 0.07						0.03					
County (ref = Oslo/Viken)	− 0.70						0.02					
*Living conditions*												
Married/cohabitate	Ref						Ref					
Single/divorced, widow/widower	0.05						− 0.12*					
*Education*												
Less than 13 years of education	− 0.09						0.06					
University less than 4 years	− 0.05						0.04					
University 4 years or more	Ref						Ref					
*Employment status (ref* = *Not paid work)*												
Full time	0.68*						0.34*					
Part time	0.54*						0.20*					
*Absence from work (ref* = *0 days)*												
1–4 days	− 0.09*						− 0.02					
5–7 days	− 0.00						− 0.04					
8–10 days	− 0.005						− 0.07					
*Household income (NOK)*												
Less than 250.000	Ref						ref					
250.000–450.000	0.12						0.03					
451.000–750.000	− 0.02						0.05					
751.000–1.000.000	0.02						0.17					
More than 1.000.000	0.10						0.24					
Self-efficacy^d^		0.21*						0.31*				
Self-esteem^e^			0.54*									
						0.54*						
*Pain (ref* = *none)*												
Less than 3 months				− 0.19*						− 0.08		
More than 3 months				− 0.50*						− 0.21*		
Loneliness^f^					− 0.015						− 0.49*	
Stress^g^						0.26*						− 0.73*
R^2^ adj^c^	23.9%	4.2%	2.6%	20.6%	2.0	6.4%	10.6%	9.4%	28.8%	3.3%	23.6%	53.8%

*P ≤ 0.05

^a^Linear regression analyses were performed separately for the RAND36 PCS and MCS as the dependent variables

^b^The independent variables were grouped into six blocks: B1–B6

^c^The strength of the associations is described in terms of standardized regression coefficients and adjusted R^2^

^d^Self-efficacy: range 1–4, where higher values indicate higher levels of self-efficacy

^e^Self-esteem: range 1–4, where higher values indicate higher levels of self-esteem

^f^Loneliness: range 8–32, where higher values indicate higher levels of loneliness

^g^Stress: range 0–1, where higher values indicate higher levels of stress

### Adjusted associations between demographic variables, psychosocial variables, pain and HRQOL

Table 5 shows the strength of the adjusted associations from the hierarchical regression analyses between the selected covariates and the dependent variables (PCS and MCS) described in terms of effect sizes and proportion of explained variance. In the final model (model 6), working part-time and, even more favourably, full time (reference: not working) had the strongest positive effects on PCS. By contrast, absence from work more than 10 days during the previous 3 months (reference: no absence from work), having pain (reference: no pain), low self-esteem, and stress had the strongest negative effects on PCS. Although not the strongest effect, living in the southern part of Norway was associated with lower scores on the PCS compared with living in the Eastern part.

**Table 5:** Adjusted associations between gender, demographic variables, psychosocial variables, pain and HRQOL examined by hierarchical regression analyses^ab^, N = 561

	SF-36 PCS						SF-36 MCS					
	M1	M2	M3	M4	M5	M6	M1	M2	M3	M4	M5	M6
Gender (Ref = father)	− 0.03	− 0.03	− 0.03	− 0.03	0.00	0.00	− 0.07	− 0.04	− 0.04	− 0.04	− 0.05	− 0.04
Age	− 0.07	− 0.06	− 0.06	− 0.06	− 0.05	− 0.06	0.03	0.04	0.03	0.02	0.06	0.00
County (ref = Oslo/Viken)	− .05	− 0.05	− 0.05	− 0.07	− 0.07	− 0.08*	0.03	0.06	0.04	0.04	0.05	0.01
*Living conditions (ref* = *Married/cohabitat)*												
Single/divorced, widow/widower	0.08	0.07	0.07	0.07	0.07	0.07	− 0.08	− 0.11*	− 0.08	− 0.07	− 0.04	− 0.03
*Education (ref* = *university 4 years or more)*												
Less than 13 years of education	− 0.06	− 0.05	− 0.05	− 0.06	− 0.06	− 0.08	0.08	0.11*	0.11*	0.10*	0.08	− 0.01
University less than 4 years	− 0.03	− 0.03	− 0.03	− 0.03	− 0.03	− 0.03	0.05	0.06	0.08*	0.08*	0.07*	0.03
*Employment status (ref* = *Not paid work)*												
Part time	0.50*	0.46*	0.46*	0.41*	0.41*	0.40*	0.14*	0.10	0.13*	0.06	0.08	0.04
Full time	0.60*	0.58*	0.58*	0.52*	0.52*	0.52*	0.26*	0.19*	0.07	0.19	0.14*	0.10
*Absence from work (Ref* = *0 days)*^c^												
1–4 days	− 0.12*	− 0.12*	− 0.12*	− 0.11*	− 0.11*	− 0.10*	− 0.06	− 0.05	− 0.03	− 0.03	− 0.01	0.00
5–7 days	− 0.10	− 0.01	− 0.01	− 0.01	− 0.01	− 0.01	− 0.05	− 0.05	− 0.04	− 0.05	− 0.06	− 0.03
8–10 days	− 0.60	− 0.06	− 0.06	− 0.05	− 0.05	− 0.05	− 0.08*	− 0.08*	− 0.07	− 0.07	− 0.06	− 0.04
More than 10 days	− 0.28*	− 0.27*	− 0.28*	− 0.25*	− 0.25*	− 0.24*	− 0.25*	− 0.24*	− 0.20*	− 0.20*	− 0.20*	− 0.13*
*Household income (NOKkr)(ref* < *250.000)*												
250.000–450.000	− 0.11	− 0.10	− 0.10	− 0.10	− 0.10	− 0.10	0.04	0.08	0.04	0.05	0.07	0.09
451.000–750.000	0.00	0.01	0.02	− 0.04	− 0.04	− 0.04	0.07	0.12	0.07	0.08	0.07	0.10
751.000–1.000.000	0.07	0.07	0.08	0.04	0.04	0.04	0.21	0.23	0.17	0.18	0.16	0.18
More than 1.000.000	0.14	0.14	0.15	0.05	0.05	0.06	0.28	0.31	0.18	0.19	0.15	0.20
Self-efficacy^d^		0.05	0.06	0.03	0.03	0.02		0.26*	0.10*	0.10*	0.07	0.07
Self-esteem^e^			− 0.02	− 0.06	− 0.06	− 0.10*			0.43*	0.44*	0.32*	0.11*
Pain (Ref = none)												
Less than 3 months				− 0.14*	− 0.14*	− 0.14*				0.04	0.05	0.08*
More than 3 months				− 0.39*	− 0.39*	− 0.37*				− 0.04	− 0.02	0.07*
Loneliness^f^					− 0.01	0.02					− 0.28*	− 0.08*
Stress^g^						− 0.10*						− 0.58*
R^2^ adj^c^	30.0%	31%	30.9%	42.3%	42.2%	42.6%	16.2%	22.1%	36.8%	37.1%	42.4%	58.7%

*P ≤ 0.05

^a^Hierarchical regression analyses were performed separately for RAND36 PCS and MCS as dependent variables

^b^The independent variables were entered into the regression in six steps, leading to six linear regression models (M1–M6)

^c^The strength of the associations is described in terms of standardized regression coefficients and adjusted R^2^

^d^Self-efficacy: range 1–4, where higher values indicate higher levels of self-efficacy

^e^Self-esteem: range 1–4, where higher values indicate higher levels of self-esteem

^f^Loneliness: range 8–32, where higher values indicate higher levels of loneliness

^g^Stress: range 0–1, where higher values indicate higher levels of stress

High self-esteem had the strongest positive effect on MCS. Stress and absence from work for more than 10 days in the past three 3 months (reference: no absence) had the strongest negative effects. Given the analysed variables, the final model explained 59% of the overall variance for MCS and 43% for PCS.

## Discussion

In this study, we aimed to identify possible associations between gender, demographic and psychosocial variables, pain and HRQOL in parents of 14–15-year-old adolescents. Most of the participants were mothers, although we recruited a sufficient number of fathers to make valid inferences about a possible role of gender. In general, mothers had lower HRQOL and reported worse psychosocial status than fathers. This finding is consistent with previous studies showing that women report lower HRQOL and worse scores for most psychosocial variables compared with men [31, 43]. However, we note that the importance of gender for HRQOL was no longer statistically significant in the adjusted analyses, which suggests that other variables such as work affiliation, pain, self-esteem, and stress were more important predictors of HRQOL in our sample.

Work affiliation had the strongest positive effect on physical HRQOL, and more frequent absence from work had the strongest negative effect. Absence from work also had a strong negative effect on mental HRQOL. A strong work affiliation is important and may reflect a feeling of commitment and desire to contribute to society, along with the self-respect paid work brings. Paid work implies income, sustenance, and safety [44] and may be considered an important contributor to the commitment aspect of being a role model for the adolescent. Most parents in our study had a university degree and a relatively high household income, which may also have affected the results. Although not identified in similar studies of parents of adolescents, being in paid work has been identified as important to HRQOL in the general population, whereas absence from work, possibly because of health problems, is considered to have the opposite effect [4, 5, 43, 44].

Stress had a strong negative effect on the parents’ mental and physical HRQOL. Previous studies have shown that parental subjective mental health status correlates significantly with the parent–child relationship and financial resources. Parental subjective physical health status is also strongly associated with more positive self-perception in adolescents [45]. Work stress and imbalance between the work and family/personal lives have been found to increase mental health problems in the working population [46]. According to Lazarus and Folkman, psychological stress refers to a person’s relationship with the environment that he or she appraises as significant for well-being and in which the demands tax or exceed available coping resources [47]. Exposure to psychosocial stressors is associated with increases in both adverse mental health outcomes and inflammatory markers [48]. Stress over time and maladapting to stressful environments might therefore have serious consequences and may lead to a condition named “allostatic overload”, which has been defined as *“the wear and tear on the body and brain resulting from chronic overactivity or inactivity of physiological systems that are normally involved in adaptation to environmental challenge”* [49]. A systematic review by Guidi et al. showed that allostatic load and overload in general are associated with poorer health outcomes [50]. The behaviour of people outside the family, such as the adolescent’s friends and their parents, and other parents in the neighbourhood, can undermine or strengthen the impact of parents on their adolescents. Therefore, parenting should be considered within a broader context, and researchers and practitioners should focus on understanding how forces outside the family accentuate or undermine the impact of parenting on adolescent development [51].

Stress had the strongest negative effect on mental HRQOL, and pain was one of the variables with a strong negative effect on physical HRQOL. Pain is a common health problem and may be a significant burden that influences both parents and their families in different ways [12, 52]. We found a high prevalence of persistent pain (> 3 moths) in the parents. This finding is consistent with earlier studies of pain in the general population that reported an association between reduced HRQOL and experiencing pain [52]. One possible explanation of the strong negative effect of pain on the physical dimensions of HRQOL is that having persistent pain may affect daily activities such as the ability to exercise and participate in social activities [12, 53]. These activities are important to the role as a parent of children at this age. Notably, a high percentage of the parents in this study (41%) reported family pain. Earlier research has shown that persistent pain in parents may influence pain attitudes and coping in adolescents and that persistent pain in parents is associated with pain in adolescents [10, 54].

### Strengths and limitations

Although the response rate was low (34%), one strength of the study is the large number of parents included. Another strength is the high number of variables included, which give a good overview of potential predictors of HRQOL in parents of adolescents. These strengths are supported by the explained variances of HRQOL of 43% for PCS and 59% for MCS in the final multivariate models. Another strength is that all variables were assessed using validated questionnaires and measures, which have favourable Cronbach α values [40].

One limitation of this study is the cross-sectional nature, which reveals only statistically significant associations between the variables and does not allow one to draw conclusions about causality. The characteristics of the parents, which included mainly mothers, and married/cohabiting and well-educated adults with a high household income, limit the ability to generalize our findings to the entire population of Norwegian parents. The small number of parents from the lower socio-economic classes and the low overall response rate study may have introduced selection bias because of the high proportion of parents who did not participant in the survey.

### Implications and future research

Overall, this study contributes to knowledge about how socio-demographic factors, pain, and psychosocial factors (self-efficacy, self-esteem, loneliness, and stress) are related to HRQOL in parents of 14–15-year-old adolescents with high socio-economic status in the general Norwegian population. This knowledge may help to inform policymakers, politicians, and health-care professionals about prioritizing and guiding the parents of adolescents. The stress reported by parents may reflect the parents’ experience during the adolescent phase, and assistance in helping parents cope with stress may help to improve their HRQOL. The high proportion of parents reporting pain and the strong association between pain and HRQOL suggest that more attention should be paid to pain and pain management, and to the potential negative effects of unemployment, not being in paid work, or sick leave/disability pension.

For future research, we suggest the use of longitudinal designs to explore our findings more thoroughly. Future research should aim to include parents with lower socio-economic status and a higher proportion of fathers. Future studies should also control for other possible confounders and add more health-related data (e.g., about exercise).

## Conclusion

Mothers reported significantly lower scores on self-efficacy, self-esteem, and HRQOL, and experienced greater stress than fathers in our sample of parents of 14–15-year-old adolescents from the general population. A high number of parents reported pain. Pain, stress, and low work affiliation were strongly associated with decreased HRQOL in parents. From the health promotion perspective, general practitioners and other health-care professionals should be aware of these predictive factors or contributors to HRQOL in parents of adolescents.

## Data Availability

The datasets used and/or analyzed during the current study are not publicly available due to General Data Protection Regulation laws but are available from the corresponding author on reasonable request and with permission from the Norwegian Centre for Research Data.

## Abbreviations

BPI: Brief pain inventory
GSE: General self-efficacy scale
HRQOL: Health-related quality of life
LPQ: Lübeck pain-screening questionnaire
OTC analgesics: Over-the-counter analgesics
PSQ: Perceived stress questionnaire
QOL: Quality of life
RSES: Rosenberg self-esteem scale
SES: Socioeconomic status
SUS: Pain, youth and self-medication study
ULS: UCLA loneliness scale
WHO: World health organization

## References

[1] Sustainable Goals. https://www.un.org/sustainabledevelopment/

[2] Sorensen J Fau - Davidsen M, Davidsen M Fau - Gudex C, Gudex C Fau - Pedersen KM, Pedersen Km Fau - Bronnum-Hansen H, Bronnum-Hansen H: Danish EQ-5D population norms. (1403–4948 (Print)).

[3] Hanmer J, Lawrence WF, Anderson JP, Kaplan RM, Fryback DG (2006). Report of nationally representative values for the noninstitutionalized US adult population for 7 health-related quality-of-life scores. Med Decis Making.

[4] Arvidsson S, Arvidsson B, Fridlund B, Bergman S (2008). Health predicting factors in a general population over an eight-year period in subjects with and without chronic musculoskeletal pain. Health Qual Life Outcomes.

[5] Wahl AK, Rustoen T, Hanestad BR, Lerdal A, Moum T (2004). Quality of life in the general Norwegian population, measured by the Quality of Life Scale (QOLS-N). QualLife Res.

[6] Bergman S, Jacobsson LTH, Herrström P, Petersson IF (2004). Health status as measured by SF-36 reflects changes and predicts outcome in chronic musculoskeletal pain: a 3-year follow up study in the general population. Pain.

[7] Perneger TV, Combescure C, Courvoisier DS (2010). General population reference values for the french version of the euroqol EQ-5D health utility instrument. Value Health.

[8] Spilker B (1996). Quality of life and pharmacoeconomics in clinical trials.

[9] Erikson EH (1965). Childhood and society, Rev.

[10] Skarstein S, Bergem AK, Helseth S (2020). How do mothers of adolescents with chronic pain experience their own quality of life?. BMC Psychol.

[11] Nygren K, Bergstrom E, Janlert U, Nygren L (2012). Parents matter–but relations to parents do not explain gender differences in self-reported health in adolescents. Scand J Caring Sci.

[12] Sylwander C, Larsson I, Andersson M, Bergman S (2020). The impact of chronic widespread pain on health status and long-term health predictors: a general population cohort study. BMC Musculoskelet Disord.

[13] Hoftun GB, Romundstad P, Rygg M (2013). Association of parental chronic pain with chronic pain in the adolescent and young adult family linkage data from the HUNT study. JAMA Pediatr.

[14] Nystrand C, Ssegonja R, Sampaio F (2019). Quality of life and service use amongst parents of young children: results from the children and parents in focus trial. Scand J Public Health.

[15] Simons LG, Conger RD (2007). Linking mother–father differences in parenting to a typology of family parenting styles and adolescent outcomes. J Fam Issues.

[16] Mastrotheodoros S, Van der Graaff J, Deković M, Meeus WHJ, Branje SJT (2019). Coming closer in adolescence: convergence in mother, father, and adolescent reports of parenting. J Res Adolesc.

[17] Calvano C, Warschburger P (2018). Quality of life among parents seeking treatment for their child's functional abdominal pain. Qual Life Res.

[18] Logan DE, Curran JA (2005). Adolescent chronic pain problems in the school setting: exploring the experiences and beliefs of selected school personnel through focus group methodology. J Adolesc Health.

[19] Mikkelsen HT, Haraldstad K, Helseth S, Skarstein S, Småstuen MC, Rohde G (2020). Health-related quality of life is strongly associated with self-efficacy, self-esteem, loneliness, and stress in 14–15-year-old adolescents: a cross-sectional study. Health Qual Life Outcomes.

[20] Dale AL KT, Roed H, Hodne T, Vangstad A. Regional monitor. In: FoU-rapport. vol 2; 2011.

[21] Cleeland CS, Ryan KM (1994). Pain assessment: global use of the brief pain inventory. Ann Acad Med Singapore.

[22] Roth-Isigkeit A, Thyen U, Raspe HH, Stöven H, Schmucker P (2004). Reports of pain among German children and adolescents: an epidemiological study. Acta Paediatr.

[23] Klepstad P, Loge JH, Borchgrevink PC, Mendoza TR, Cleeland CS, Kaasa S (2002). The Norwegian brief pain inventory questionnaire: translation and validation in cancer pain patients. J Pain Symptom Manage.

[24] Haraldstad K, Sørum R, Eide H, Natvig GK, Helseth S (2011). Pain in children and adolescents: prevalence, impact on daily life, and parents' perception, a school survey. Scand J Caring Sci.

[25] Skarstein S, Rosvold EO, Helseth S, Kvarme LG, Holager T, Småstuen MC, Lagerløv P (2014). High-frequency use of over-the-counter analgesics among adolescents: reflections of an emerging difficult life, a cross-sectional study. Scand J Caring Sci.

[26] Kvien TK, Kaasa S, Smedstad LM (1998). Performance of the Norwegian SF-36 health survey in patients with rheumatoid arthritis. II. A comparison of the SF-36 with disease-specific measures. J Clin Epidemiol.

[27] Ware JE, Kosinski MA, Keller SD (1994). SF-36 Physical and Mental health summery scale: a user’s manual.

[28] Ware JE, Snow KK, Kosinski MA, Gandek MS (1993). SF-36 health survey manual and interpretation guide.

[29] Loge JH, Kaasa S, Hjermstad MJ, Kvien TK (1998). Translation and performance of the Norwegian SF-36 health survey in patients with rheumatoid arthritis. I. Data quality, scaling assumptions, reliability, and construct validity. J Clin Epidemiol.

[30] Luszczynska A, Scholz U, Schwarzer R (2005). The general self-efficacy scale: multicultural validation studies. J Psychol.

[31] Bonsaksen T, Lerdal A, Heir T, Ekeberg Ø, Skogstad L, Grimholt TK, Schou-Bredal I (2019). General self-efficacy in the Norwegian population: Differences and similarities between sociodemographic groups. Scand J Public Health.

[32] Rosenberg T (1965). Society and the adolescent self-image.

[33] Tambs K, Røysamb E (2014). Selection of questions to short-form versions of original psychometric instruments in MoBa. Norsk epidemiologi.

[34] Hays RD, DiMatteo MR (1987). A short-form measure of loneliness. J Pers Assess.

[35] Russell D, Peplau LA, Cutrona CE (1980). The revised UCLA Loneliness Scale: concurrent and discriminant validity evidence. J Pers Soc Psychol.

[36] Process of translation and adaptation of instruments. https://www.who.int/substance_abuse/research_tools/translation/en/

[37] Levenstein S, Prantera C, Varvo V, Scribano ML, Berto E, Luzi C, Andreoli A (1993). Development of the perceived stress questionnaire: a new tool for psychosomatic research. J Psychosom Res.

[38] Kocalevent RD, Levenstein S, Fliege H, Schmid G, Hinz A, Brähler E, Klapp BF (2007). Contribution to the construct validity of the perceived stress questionnaire from a population-based survey. J Psychosom Res.

[39] Østerås B, Sigmundsson H, Haga M (1850). Psychometric properties of the perceived stress questionnaire (PSQ) in 15–16 years old Norwegian Adolescents. Front Psychol.

[40] Altman DG (2006). Practical statistics for medical research.

[41] Cohen J (1988). Statistical power analysis for the behavioral sciences.

[42] Pallant J (2016). SPSS survival manual: a step by step guide to data analysis using IBM SPSS.

[43] Garratt AM, Stavem K (2017). Measurement properties and normative data for the Norwegian SF-36: results from a general population survey. Health Qual Life Outcomes.

[44] Livskvalitet i Norge 2020 - Quality of Life in Norway 2020. https://www.ssb.no/sosiale-forhold-og-kriminalitet/artikler-og-publikasjoner/_attachment/433414?_ts=174f89c29d0

[45] Giannakopoulos G, Dimitrakaki C, Pedeli X, Kolaitis G, Rotsika V, Ravens-Sieberer U, Tountas Y (2009). Adolescents' wellbeing and functioning: relationships with parents' subjective general physical and mental health. Health Qual Life Outcomes.

[46] Wang JL (2006). Perceived work stress, imbalance between work and family/personal lives, and mental disorders. Soc Psychiatry Psychiatr Epidemiol.

[47] Lazarus RS, Folkman S, Appley MH, Trumbull R (1986). Cognitive theories of stress and the issue of circularity. Dynamics of stress.

[48] Maldonado A, Preciado A, Buchanan M, Pulvers K, Romero D, D'Anna-Hernandez K (2018). Acculturative stress, mental health symptoms, and the role of salivary inflammatory markers among a Latino sample. Cultur Divers Ethnic Minor Psychol.

[49] McEwen BS (1998). Stress, adaptation, and disease. Allostasis and allostatic load. Ann N Y Acad Sci.

[50] Guidi J, Lucente M, Sonino N, Fava GA (2021). Allostatic load and its impact on health: a systematic review. Psychother Psychosom.

[51] Steinberg L (2001). We know some things: parent-adolescent relationships in retrospect and prospect. J Res Adolesc.

[52] Andrews P, Steultjens M, Riskowski J (2018). Chronic widespread pain prevalence in the general population: A systematic review. Eur J Pain.

[53] Zadro JR, Nilsen TIL, Shirley D, Amorim AB, Ferreira PH, Mork PJ (2018). Parental chronic widespread pain and the association with chronic widespread pain in adult offspring: family-linkage data from the Norwegian HUNT Study. Eur J Pain.

[54] Hoftun GB, Romundstad PR, Rygg M (2013). Association of parental chronic pain with chronic pain in the adolescent and young adult: family linkage data from the HUNT Study. JAMA Pediatr.

